# Supplementary material for: Bacterial Microbiota in Soil Amended with Deoxynivalenol-Contaminated Wheat
Source: Toxins (Basel). 2025 Nov 22;17(12):565. doi: 10.3390/toxins17120565 (PMC12737705; doi:10.3390/toxins17120565)
Supplement: Supplementary file 1 [file toxins-17-00565-s001.zip › toxins-3961857-supplementary.pdf]

Supplementary Materials: Bacterial Microbiota in Soil Amended with Deoxynivalenol-Contaminated Wheat

Table S1. Chemical analysis of soil samples.

**Sample Type**	**Subsample**	**Moisture content, dwb %**	**NO_3-N mg/kg**	**NH_4-N, mg/kg**	**pH in $CaCl_2$**	**pH in H_2O**	**EC in H_2O, uS cm^{-1}**	**Total N, mg N g^{-1}**	**Total C, mg C g^{-1}**	**C/N (calculated)**	**Total Organic Carbon, mg C g^{-1}**	**TOC/TN (calculated)**	**$\delta^{12}N$, ‰**	**$\delta^{13}C$, ‰**	**$\delta^{13}OC$, ‰**
Manure	A	14.2%	3.9	1.0	6.9	7.3	241	2.5	28.5	11.2	23.7	9.4	9.3	-20.5	-23.4
Manure	B	14.9%	2.9	0.91	6.7	7.3	222	2.5	30.7	11.9	26.0	10.1	9.7	-21.1	-23.6
Manure	C	14.8%	3.6	0.82	6.9	7.3	234	2.4	28.7	11.6	24.7	10.0	9.6	-20.7	-23.5
Non manure	A	12.8%	0.9	0.68	7.0	7.2	168	1.8	21.2	11.7	17.9	9.9	7.1	-20.5	-23.2
Non manure	B	13.5%	1.2	0.87	7.1	6.4	158	1.7	21.2	11.9	18.1	10.1	6.9	-20.6	-23.2
Non manure	C	14.1%	1.5	0.73	7.0	7.4	159	1.8	22.3	11.9	18.6	10.0	7.0	-20.8	-23.3
Lacombe	A	18.2%	12.2	0.80	4.8	6.4	133	3.3	38.7	11.7	*		6.3	-25.7	*
Lacombe	B	18.0%	13.9	0.79	5.2	6.3	141	3.3	38.8	11.6	*		6.2	-25.6	*
Lacombe	C	18.8%	13.8	0.81	5.1	6.4	144	3.3	38.3	11.6	*		6.1	-25.7	*

* The limited inorganic C in Lacombe soil did not allow detection of a difference between %TOC and %TC; therefore the same values for %TC and d13C were assumed for %TOC and d13OC.
